# How Does the Presence of Others Influence Control Inhibition? Contradictory Evidence Using an Antisaccade and Stop Signal Task

**DOI:** 10.1177/00332941231153328

**Published:** 2023-01-19

**Authors:** Teresa Garcia-Marques, Alexandre Fernandes

**Affiliations:** William James Center of Research, ISPA-Instituto Universitário, Lisbon, Portugal

**Keywords:** Executive control, inhibition, presence of others

## Abstract

Inhibitory control (IC) is defined as the (in)ability to change, suppress, or delay a response that is no longer required under the current circumstances. This ability was previously argued to increase in social contexts, based on Stroop’s performance, showing that participants performed the Stroop task better in others’ presence than alone. In this paper, we extend the testing of this same hypothesis to the use of two other tasks that Mitake et al. (2000) show to grasp the same IC ability; the Antisaccade and Stop signal tasks. If Stroop’s performance was capturing the impact of the presence of others on CI abilities, the effect would generalize to performance on these tasks. This hypothesis was only generally supported by stop signal task performance; those in the presence condition were significantly more efficient than those in the alone conditions. For the Antisaccade tasks, evidence shows that higher levels of interference occurs in the presence of others condition for participants' fastest responses We discuss how this evidence contributes to the literature suggesting that the two tasks may index different constructs.

## Introduction

Inhibitory control (IC) is defined as “the (in)ability to change, suppress or delay a response that is no longer required under the current circumstances” (e.g., Logan & Cowan, 1984). This IC is the hallmark of executive control (e.g., Bickel et al., 2012; Miyake et al., 2000). The IC defines the ability of individuals to exert control over their automatic responses, suppressing them, when necessary, in favor of an alternative response.

IC ability has been argued to increase in social contexts. This claim has been sustained in the fact that compared with a condition where participants performed the Stroop task alone, the performance in a Stroop task in the presence of others was better (e.g., Fernandes et al., 2021; Huguet et al., 1999). New Stroop studies have now replicated this evidence (see Garcia-Marques & Fernandes, under review, for a metanalytic summary of the effect) adding to strength to the suggestion that when the ability to inhibit control is operationalized as better performance on the Stroop task, participants, in the presence of others, are more effective in exerting control.

Our goal is to test the hypothesis that IC ability increases in social contexts, and at the same time to turn this test independent of an effect previously obtained with the Stroop task. As such, we intend to extend the evidence that give support to this hypothesis to individuals’ performance in two other tasks. We selected the two tasks for which data from Miyake et al.’s (2000) psychometric study showed a saturation (shared variance) on the same factor as the Stroop task, presumably offering alternative measures of the same inhibition ability: the Antisaccade task (e.g., Hallett, 1978; McFall et al., 2009; Strukelj et al., 2016) and the stop signal task (Logan, 1994; Logan & Cowan, 1984).

The Antisaccade task (Hallett, 1978) allows researchers to infer participants' level of IC by attending to their ability to look opposite to that of a suddenly appearing peripheral stimulus (antisaccade trials), supposedly referring to the inhibition of the automatic eye movements toward the peripheral stimulus which is allowed in some trials (prosaccade trials). As such in the Antisaccade task participants are instructed in some of the trials to look opposite of a suddenly appearing peripheral stimulus, avoiding eye movements toward it. Then performance in these trials is compared with performance in prosaccade trials that only demand alertness and gaze reorienting (an automatic prepotent/dominant response). The correct performance on the Antisaccade task requires the ability to withhold an initiated automatic response (inhibition of the automatic prosaccade toward the stimulus) plus the generation of the antisaccade movement (Hutton et al., 2006). At a behavior level impaired antisaccade IC performance is either indexed by erroneous prosaccades or an increased reaction time (RT) to respond correctly (see e.g., Johnson et al., 2016; Miyake & Friedman, 2012; Kane et al., 2001; Todd et al., 2022). The increased correct antisaccade RTs are supposed to occur given the additional processing resources used for inhibiting the automatic prosaccades (Olk & Kingstone, 2003), so these RTs index the “control efficiency” of participants It was with these indexes of IC, that Miyake et al.’s (2000) psychometric study show commonalities with indexes from the Stroop and the Stop Signal tasks.

There are already two studies, from Oliva et al. (2017) and Tricoche et al., (2020), that provide evidence considering the performance on the Antisaccade task in and out of a social context. Both of them contradict the hypothesis that presence increases IC. Both studies assessed antisaccade performance at an oculomotor level, being IC indexed by comparing the pro and antisaccade eye movement direction error and the pro and antisaccade latency (e.g., Guitton et al., 1985; Hutton et al., 2006) and strongly differ in their manipulation of others’ presence. Oliva et al. (2017) addressed the effect of others’ presence by comparing an isolation condition with a coaction context. They had the presence condition defined by 2–8 co-actors' presence and the alone condition with only the participant in the room*.* And Tricoche et al. (2020) compared the performance of participants in a condition where a familiar partner that was completing questionnaires on a laptop seated on the right of the participants, is present in the room with a condition where participants were alone*.* This presence manipulation, unlike Oliva et al.’s (2017) lacks adequate isolation of the presence factor from others by confounding it with familiarity (see Bond & Titus, 1983; Guerin, 1993). Additionally, unlike Oliva et al. (2017) and Tricoche et al. (2020) assessed the impact of having participants perform a complex or a simple Antisaccade task.

The two studies show different results. Oliva et al. (2017) results show that the presence of co-actors in the room impairs antisaccade performance, while prosaccade performance remained the same. Authors claim that this occurred due to an increased difficulty of participants in others’ presence to suppress the automatic prosaccades. This claim is apparently contrary to our hypothesis and so to what one would expect from the studies focusing on Stroop performance (e.g., Huguet et al., 1999) if the performance in the Antissacade task was reflecting inhibition abilities. In this study, the evidence provided by saccadic latency data shows that in the presence of others the antisaccade responses are always slower and that for the participant to provide correct responses, they require an extra delay. In their second experiment results showed only evidence of the main effect of presence on the general performance being the effect stronger for higher numbers of co-actors than a lower number. Tricoche et al.’s (2020) results regarding oculomotor responses showed that the presence of a familiar partner had an impact on subjects' overall performance, but that this impact depended on whether the task was complex (antisaccades were mixed pseudorandomly) or simple (antisaccades were performed separately); presence of others exerted a negative impact on participants' overall performance (for both pro- and antisaccade trials) when the task was complex and presence of others exerted a positive impact on participants' overall performance when the task was simple. Saccade RTs (responses, speed to initiate the saccade, and speed to move the eye toward the target) show the main effect of the presence of the familiar partner also to be moderated by the type of task. But there were no interaction effects with the nature of the task and so no evidence that the presence differently reduces the saccade RTs for antisaccade more than for prosaccades, which would represent an increase in IC. As such the data of this study offer no evidence regarding IC abilities.

Taken together, these two studies suggest as likely that results from an Antisaccade task will not corroborate the conclusions that were taken from data obtained with the Stroop task. This increases the relevance of approaching the hypothesis regarding if and how presence modulates IC abilities, using the same procedure that Miyake et al. (2000), have previously shown to provide IC ability measures congruent with a Stroop task and the Stop Signal task.

To our knowledge, no previous study contrasted participants’ performance in a Stop Signal task between the conditions alone to mere presence or to coaction. This task grasps the individuals’ ability to withhold an already initiated response plus the generation of another response (Logan, 1994; Logan & Cowan, 1984). In this task, a prepotent/dominant response is a motor response and is established through learning. promoting speeded reaction times to an arbitrary cue on most of the trials. Because trials and responses are uninterrupted, the prepotent or dominant response is reinforced. In a small number of trials, a “stop signal” cue is presented, prompting participants to detect that their prepotent motor response will be an error. Participants overcome the dominant response by delaying their response. Thus, the stop signal correct response RTs provide information about the IC ability of individuals (see Band et al., 2003). We expect to find evidence supporting the hypothesis that this ability is higher for those performing the task in a co-action setting than it is in isolation.

Strategically in our studies, we use the versions of these two tasks previously defined by Miyake et al. (2000) in their psychometric study of the measurement of executive control functions. Thus, the *Antisaccade task* was adapted from Roberts et al. (1994), and the *stop-signal task* was adapted from Logan (1994). As already stated, we use these tasks and their measures because, in Miyake et al. (2000), they were shown to load highly and reliably on the inhibition function together with the classic Stroop task. Both of these tasks offer an index of CI that results from comparing a performance characteristic (RT or number of correct answers) on trials of a different nature. In addition to assessing the impact of others’ presence over these indexes of IC on both tasks, we also analyze their temporal features with a delta-plot analysis (see Ridderinkhof, 2002). In Sharma et al. (2010) this delta-plot analysis turned clear that the evidence of an increased control promoted by others' presence in Stroop performance, occurs only for longer responses. As such this analysis will allow determining whether the detection of inhibition efficiency promoted by the presence of others, occurred either for faster (likely due to mapping interference at earlier processing mechanisms) or for longer response times (likely mapping some later processing mechanism, conceptually replicating previous findings, Sharma et al., 2010; see also Fernandes et al., 2021; Garcia-Marques et al., 2015).

## Experiment 1: Antisaccade Task

### Method

#### Participants

Participants were recruited from a pool of subjects (managed by the University Psychology Laboratory) composed mostly of students from universities in the same city. We targeted 72 participants under 30 years of age.^1^ The sample size was estimated based on the effect sizes of the Antisaccade task studies reported above and those studies that assessed the effects of the presence of others in inhibition tasks, particularly the most studied Stroop task (*f* = .15, *α* = .05, 1 − *β* = .90; Faul et al., 2007). Participants were all native Portuguese-speaking (65 females, Mean age = 21.30, SD = 3.02). The following methods were approved by the University Ethics Review Board and conducted in accordance with the Declaration of Helsinki. Participants gave informed consent before the study and received payment (5 euros) for their participation.

#### Design and materials

The *Antisaccade task* was programmed in E-Prime 2.0 software (Schneider et al., 2002), and the stimuli were presented on a 24-inch monitor with a resolution of 1.920 × 1.080 pixels (144 Hz) against a white background.

**Figure 1.**
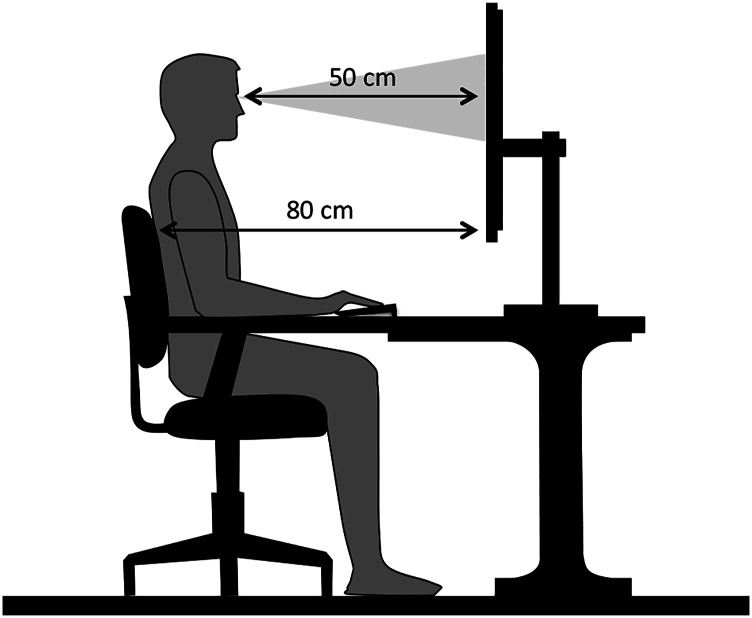
Individual setup, marking distances to the computer screen.

Support between the table and the chair made it possible to keep the back of the chair at a fixed distance from the monitor. The participant was asked to always remain leaning with the back straight so that the distance from the monitor was approximately 50 cm, in order to maintain the relative size of the perceptual elements (see Figure 1), being within the range used in antisaccade studies (Holmqvist et al., 2011).

Variables were manipulated between factorial design composed of 2 (saccade: prosaccade vs. antisaccade) x 2 (presence: alone vs. co-action) conditions. In the presence condition, the participants performed the task simultaneously with other participants in the same room, while in the alone condition, they performed it in isolation.

#### Procedure

**Figure 2.**
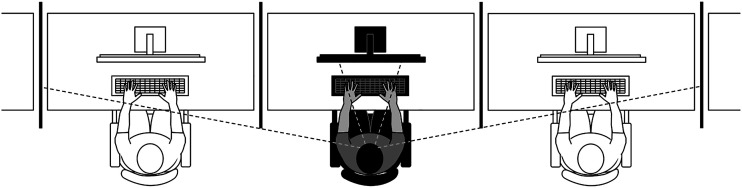
Experimental setting for manipulating the presence of other conditions.

Participants executed the task in a room equipped with 5 computers on separate tables with chairs 1.5 m apart; screens were placed between the tables to prevent participants from seeing the computer monitors and the performance of other participants. In this setting, participants would be aware of the presence of others through peripheral vision and hearing (see Figure 2). Participants either performed the task in a presence setting (i.e., groups of 4–5 participants doing the same task without competing or interacting) or alone in the same room. In both conditions, the experimenter informed participants that he/she would be in another adjacent room, where they should meet him/her at the end of the task if he/she had not returned in the meantime. The presence condition had 36 participants, and the alone condition had 36 participants.

**Figure 3.**
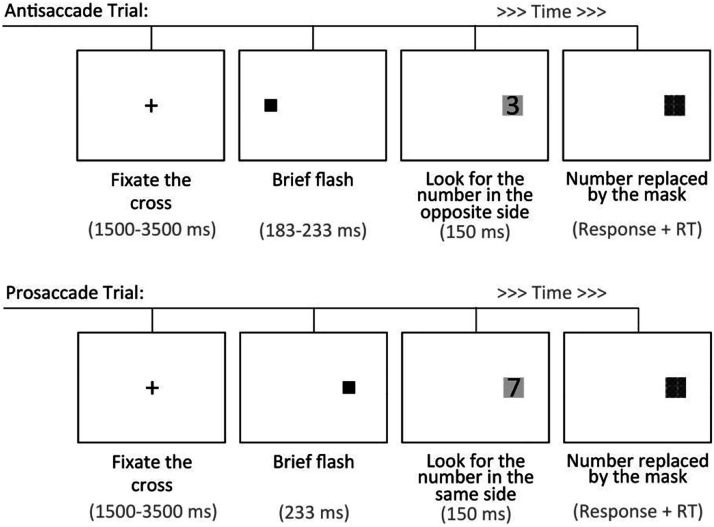
Upper panel: Timeline of an antisaccade trial. Bottom panel: Timeline of a prosaccade trial.

The Antisaccade task had each trial starting with a fixation cross that was displayed for a duration of times varying over 9 levels (1500–3500 ms at 250-ms intervals). Then, an initial cue (0.3-cm black square - 0.3°) was presented at 8.6 cm - 10° - (inner edge) to the left or right of the fixation cross, which preceded the appearance of a target (digits 1–9) inside a gray square (of a 1.11-cm size - 1.3°) at a distance of 8.26 cm (inner edge) from the screen center. The target remained visible for 150 ms until it was masked by a crosshatched gray square (see Figure 3). In each trial, the participant reported (or guessed) which target number was displayed (using the keyboard), and the response time was also recorded.

In an initial prosaccade block with 18 trials, the target was always presented (233 ms after the cue) on the same side as the cue. This presentation had the goal of building a prepotency to orient to the same side of the cue. Three antisaccade blocks were followed, each containing 36 trials in which the target always appeared on the opposite side of the cue. The cue-to-target interval was fixed at 233 ms for the first block, 200 ms for the second block, and 183 for the third block. The stimuli were pseudorandomly ordered so that each target number (and fixation duration) was presented at equal times on each block and side of the screen. At the beginning of the prosaccade block, there were 6 warmup training trials, and in the first antisaccade block, there were 12 warmup training trials that were not analyzed.

### Dependent Measures and Data Analysis

Correct responses**.** The main dependent measure was the number of correct keyboard responses (CR) occurring for each participant in the prosaccade and in the antisaccade blocks.

#### Reaction times (RTs) for CR

We also analyzed the reaction times (RTs) for correct responses. The correct RTs (85.83% of the data) were trimmed by replacing the values below 300 ms and above 3000 ms with these threshold values (0.40% of the data) to minimize disproportionate influences from data-points outliers.

#### Delta plots

The difference between the prosaccade and the antisaccade blocks for each participant that defined the IC ability effect was in addition analyzed for different levels of each participant’s reaction times, allowing comparing these delta plots across those conditions. These delta plots characterize the temporal dynamics of the antisaccade effect (see Unsworth et al., 2011), clarifying if the effect (represented by delta plots of the difference between prosaccade and antisaccade trials) is better detected when participants provide quick or longer responses. We use the delta plots to approach the inhibition function based on the index of RT, analyzing the IC ability effect distributions by following the same principles that were applied in other inhibition tasks, such as Stroop (de Jong et al., 1994; Ridderinkhof, 2002). To have the antisaccade effect plotted for shorter and longer responses, we first, rank-ordered individual RTs of correct and incorrect responses separately for prosaccade and antisaccade trials. Next, four successive bins of equal size (25% of responses) were created from the rank-ordered response times corresponding to each participant. Correct RT was then determined for each participant’s quartile. To calculate delta values representing the antisaccade effect for each of these quartils we first substract RTs from the antisaccade quartil from the same prosaccade quartil. For normalizing these delta values for RTs, representing the antisaccade effect (AE) we divided them by the RT mean for each quartile, (bin_prosaccade) + (bin_antisaccade)]/2 and calculated AE/RT for each quartile.

#### Statistical analyses

Data analysis was conducted using Jamovi (Şahin & Aybek, 2019). We index IC ability by the number of correct responses (CR) and their reaction times (RTs), using them as dependent measures separate ANOVAs defined by the experimental mixed design 2 (saccade: prosaccade vs. antisaccade) x 2 (presence: alone vs. coaction) having the first factor as a within-participant factor. Normalized delta plots defining the IC ability at a different level of response time distribution were analyzed using an ANOVA 4 (quartile distribution bins) x 2 (presence: alone vs. co-action) having the first factor as a within-participant factor.

## Results

### Number of Correct Responses

The number of correct responses was analyzed within an ANOVA defined by the experimental design. Results show that the number of correct responses was different for anti- and prosaccade trials, F(1,70) = 116.43, *p* < .001, η^2^_p_ = .63 As expected, accuracy was higher for the prosaccade trials (M = .99, SE = .002) than for the antisaccade trials (M = .85, SE = .013; e.g., McFall et al., 2009; Roberts et al., 1994; Olk & Kingstone, 2003). Contrary to what would be expected, the presence of others did not affect this performance (F <1^2^; Presence condition main effect: F(1,70) = 0.4; Interaction: F(1,70) = 0.06).

### Reaction Time to Correct Responses

The RTs means associated with correct responses were analyzed within an ANOVA defined by the experimental design. Replicating previous research (e.g., McFall et al., 2009; Olk & Kingstone, 2003; Roberts et al., 1994), analysis shows a significant main effect of the type of saccade, F(1,70) = 8.11, *p* = .006, η^2^_p_ = 0.10 mm, given that RTs were shorter for the prosaccade trials (M = 815, SE = 20.3) than for the antisaccade trials (M = 854, SE = 14.9). The main effect of presence, F(1,70) = 8.64, *p* = .004, η^2^_p_ = 0.11, shows that participants are quicker to respond in the presence condition (M = 7 86, SE = 23.3) than in the alone condition (M = 883, SE = 23.3). However, there is no significant interaction between the two factors (F(1,70) = 1.27, *p* = .284, η^2^_p_ = .02).

### Delta Plots Analysis

**Figure 4.**
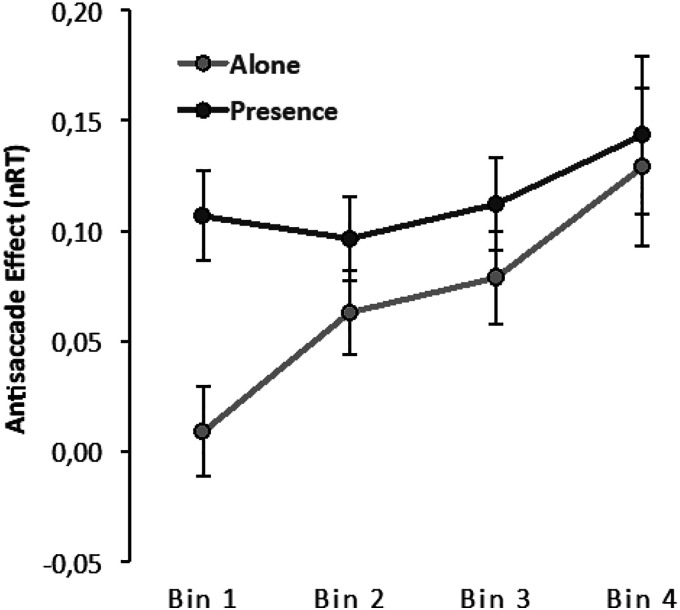
RT delta plots showing the antisaccade effect as a function of normalized RTs (i.e., nRT) for the alone and presence conditions.

As referred to above normalized delta plots for the antisaccade effect were binned in the RT quartiles at each participant level. The delta plot function defines the cost of the preparation for inhibition of the saccade in the antisaccade trials at each RT distribution level. Visual inspection of the plot (see Figure 4) shows that the function is positive, suggesting that the cost of inhibition increases with increasing response time. Analysis corroborated the significance of this pattern, F(3,210) = 9.82, *p* < .001, η^2^_p_ = .12.

For each presence condition, delta plots were compared using a 2(presence) x 4(bins) ANOVA. The analysis reveals the presence main effect, F(1,70) = 2.22 *p* = .141, 2 *p* = .03, which was previously identified in the ANOVA done directly over the RTs. However, we now learn that this factor interacted with the delta plot effect, F(3,210) = 2.98, *p* = .032, η^2^_p_ = .04, showing that there is at least one segment in the task that differs for those alone or in the presence of others. A post hoc comparison clarified that differences occurred only when participants gave faster responses; those in the presence of others started the response processes less efficiently than those alone, t(70) = 3.40, *p* = .023. The high level of interference occurring for those in the presence of others is then maintained independently of the time taken to respond. The expected increase in interference promoted by time occurred only for those in the alone condition, subsequently leading to no differences (all *p* > .70) between conditions for all other bins.

In summary, the analyses of the Experiment 1 results show evidence of the predicted inhibition effects reflected as an antisaccade effect (difference between pro and antisaccade responses) both in reaction times and accuracy. However, against our expectations, the effect was not moderated by the type of presence. Although those in the present condition were faster in their responses, this quickness did not impact how they experienced the interference in the antisaccade trials relative to those in the alone condition. This pattern of results with perceptual responses is similar to the results reported by Tricoche et al. (2020) in their ‘simple task’ when indexing IC by oculomotor responses. However, interestingly, delta-plot analysis suggests that differences in the presence condition are better detected in participants' quicker responses. The direction of this effect is the same as that obtained by Olivia et al. (2017) with oculomotor responses, likely suggesting that the effect occurring at this level is better captured in a quicker perceptual response (being less affected by other mechanisms; see general discussion). According to this, interference is probably happening faster for people in the presence condition than it is for people who are alone.

## Experiment 2: Stop-Signal Task

### Method

#### Participants

A total of 72 participants aged above 19 and below 30 years (67 females, Mean Age = 20, SD = 2.93) were recruited for this experiment. All sample selection procedures and ethical requirements for this new sample of participants were similar to the procedures and requirements followed in Experiment 1.

#### Design and materials

Variables were manipulated in a mixed factorial design composed of 2 (signal: no-signal vs. stop-signal) x 2 (presence: alone vs. presence of others) conditions. We adapted the stop-signal task as presented by Logan (1994) to our goal of preventing participants from offering only correct responses and being able to capture their response latencies for delta plot analysis. For this purpose, we capture response time baselines of individuals and calibrate the stop-signal timing to the individuals in a constant way.

A total of 296 words were selected from various standards of words in the Portuguese language (refs.) as target stimuli. These words were the most familiar words from two categories: 148 animals (e.g., bee, butterfly, horse) and 148 objects (e.g., spoon, lock, table).

#### Procedure

**Figure 5.**
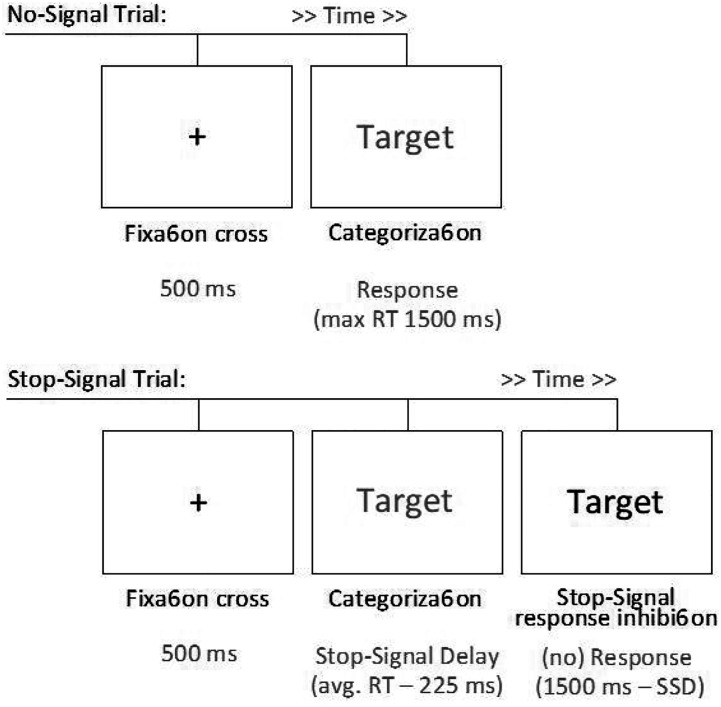
Upper panel: Timeline of a no-signal trial (or go trial). Bottom panel: Timeline of a stop-signal trial (or nogo-trial).

Participants executed the task in the same setting and conditions as used in Experiment 1 (see Figure 5). The presence condition had originally 35 participants, and the alone condition had 43 participants.

The stop-signal task consisted of five blocks of trials. The first block was composed of 48 no-signal trials used to build a prepotent categorization response. In each no-signal trial (see Figure 5), participants saw one target word (presented in green color), then had to categorize the target word as either an animal or nonanimal (i.e., object) quickly and accurately as possible. In the following four blocks of 48 trials each, participants were instructed to inhibit their categorization response in 25% of trials, which was signaled by a color change (from green to red) of the target word (see Figure 5). This leads that in the majority of trials a participant is presented with a stimulus within a green square to which they have to respond by pressing a key associated with the category animal or a key associated with the category non-animal. However, when the square changes to red (a stop signal is presented), they should avoid providing such categorization. Occasionally the stop signal (i.e., the signal to change from green to red) in those trials appeared slightly later than the stimuli. This delay (stop-signal delay: SSD) was calculated as the subtraction of 225 ms from the RT average of the participant in the no-signal trials presented in the first block. Participants were asked not to slow down the response time but to attend to the stop signal (see Logan, 1994). Each trial (with or without the stop sign) had a maximum duration of 1500 ms. It ended earlier if the participant provided a classification of the target word (a correct answer on trials without the stop sign and a wrong answer on trials with the stop sign). The onset of the stimulus occurred after a fixation cross lasting 500 ms (see Figure 9). Eight training trials were included before the no-signal block and before the first of the three stop-signal blocks (each with 36 no-signal plus 12 stop-signal trials).

### Dependent Measures and Data Analysis

The main measures were the correct responses (CR) and their reaction times (RTs). These measures allow us to study the cost of preparation for inhibition.

#### CR cost index

The comparisons of the proportion of correct responses between performance in the no-signal trials that required preparation for inhibition (stop-signal blocks) and no-signal trials that did not require preparation for inhibition (no-signal block).

#### RT cost index

These RT comparisons were computed from accurate trials only. Data from trials with RT less than 300 ms (anticipatory responses) were replaced with that threshold value. The same difference between no-signal trials from the two blocks was computed.

#### Delta-plots of RT cost index

The Rt cost index was analyzed at the individual level for each quantile of the participant RT distribution of responses. These delta plots characterize the temporal dynamics of the “antisaccade effect” (antisaccade vs. pro-saccade) calibrated at the individual level.

#### Data analysis

Data analysis was conducted using Jamovi (Şahin & Aybek, 2019) after removing five participants from our sample for not performing the full task as required (one from the alone condition and four from the presence condition). In the analysis of cost, we controlled for baseline response latencies of individuals. This is because these baselines determined the levels of SSD that participants were experiencing in the task they were performing, reflecting the level of inhibitory ability demanded by each individual. Those who were quicker to respond to the control trials were exposed to shorter delays than those who responded slowly.

As in Experiment 1, we explored the temporal dynamics of the inhibition effects for RTs (RT cost index) plotting the inhibition effect as a function of five normalized RT bins.

## Results

### RT cost of preparation for inhibition

We address the cost of preparation for inhibition at the level of RTs for those who respond in the alone versus presence conditions. The results reflect a significant effect of type of presence, t(71) = 2.11, *p* = .038, d = 0.50, occurring because those in the presence condition show evidence of having less RT costs (M = 52 ms, SD = 103) than those in the alone conditions (M = 101 ms, SD = 93). We further test if the SSD of the individual interferes with these results by adding this variable and its interaction with the presence factor as a continuous variable into a General Linear Model approach. The analysis shows that the SSD of the participant was not related his/her RT cost, F(69) = 2.12, *p* = .150, η^2^_p_ = .03, nor moderate the effect of the presence of others (interaction; F <1; F(69) = 0.07).

### Response Accuracy Cost of Preparation

**Figure 6.**
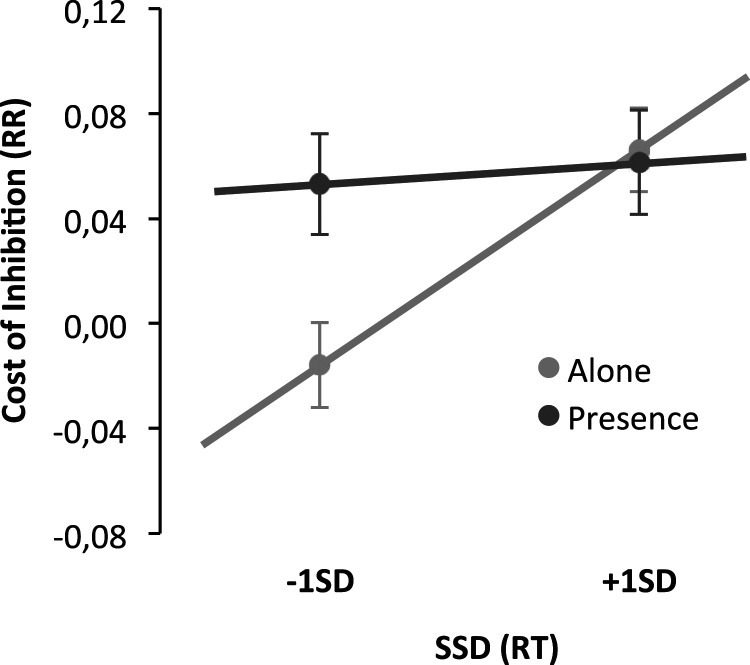
A difference in response error rate for the no stop-signal trials in the two blocks, dependent upon SSDs in the alone and presence conditions.

The difference in response error rates (RRs) in no-stop trials given within the two blocks (no-signal and stop-signal blocks) was contrasted in a General Lineal Model approach for participants in the presence and alone conditions, with SSD as a continuous variable. The results showed a nonsignificant main effect of others' presence, F(69) = 3.26, *p* = .075, η^2^_p_ = .05. The significant main effect of SSD, F(69) = 6.17, *p* = .015, η^2^_p_ = .08, was however qualified by the presence condition F(69) = 4.15, *p* = .046, η^2^_p_ = .06. This interaction emerged because the level of SSD of individuals determines performance only for those in the alone condition and not for those in the presence condition. The cost of preparation for those in the presence condition was independent of their SSDs (see Figure 6).

### Delta Plots of RT Cost of Preparation

**Figure 7.**
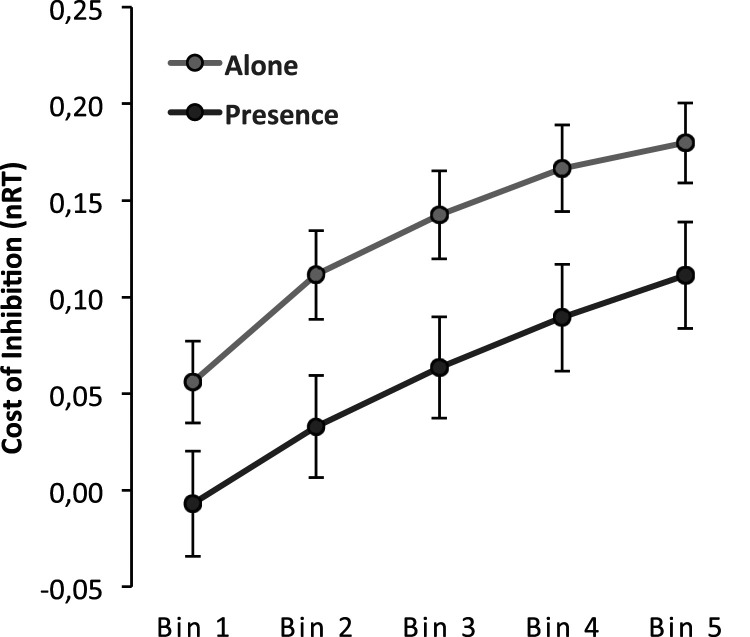
RT delta plots showing the inhibition effect as a function of normalized RTs (i.e., nRT) for the alone and presence conditions.

We calculated delta plots for the RT costs with the same procedure used in Experiment 1, allowing us to plot the timing cost of preparation against the pooled mean RT of each bin. Visual inspection of the plot (see Figure 7) shows replicating previous evidence (e.g., Khng & Lee, 2014). The delta plot is positive, showing that the cost of inhibition increases with increasing response time. Analysis corroborated the significance of this pattern, F(4,284) = 42.27, *p* < .001, η^2^_p_ = .37.

Corroborating the previous analysis, the main effect of presence is significant F(1,71) = 5.06, *p* = .028, η^2^_p_ = .07, and this suggests that the cost is higher for those in the alone condition (M = 0.13, SE = 0.02) than for those in the presence condition (M = 0.05, SE = 0.02). This effect was constant for all bins (interaction: F <1 (F(1,284) = 0.23), so that response time was not different, supporting the overcoming of the cost.

## General Discussion

The results of the two experiments do not offer congruent conclusions about the hypothesis that when in the presence of others, participants increase their IC performance. Concerning the Antisaccade task, the direct analysis of the antisaccade effect shows no evidence was found that control is exerted differently in the two conditions. With regard to the stop-signal task, the results suggest that in the presence of others and independent of individuals’ variability (SSD), participants were significantly more efficient in their inhibition than those in the alone conditions (RT analysis). Contrary to what occurred in the alone condition, for those in presence conditions, the costs of preparation over response accuracy were independent of the response-ability of the participants.

Thus, only the stop signal task offers evidence that can support the hypothesis that those in presence are more likely to efficiently inhibit cognitive interferences in their responses. This evidence corroborates the impact of the presence of others on the performance of the Stroop task, which was shown to be a reliable effect (see meta-analysis Garcia-Marques & Fernandes, under review).

However, data from the Antisaccade task offer two informative cues that establish a new challenge. One cue is that those in the presence condition were quicker in their responses than those in the alone condition. This general effect obtained with perceptual responses in our task replicates the oculomotor responses results of Tricoche et al. (2020), given that our task better matches their “simple tasks” given that pro- and antisaccades were performed separately and not pseudorandomly mixed. The other cue is that the differences between the presence conditions, which would replicate Oliva et al. (2017) results, were detected in our delta plot analysis, but only for participants' quicker responses. This may relate to the fact that Oliva et al. (2017) assessed ocular motor responses and we assessed perceptual responses; likely the pattern of the eye control RTs is more easily noticed in participants quicker than longer responses (whereas motor response control mechanisms may occur). The direction of the effect is not the one expected by the hypothesis of others' presence promoting an increase in IC abilities. What seems to suggest is that interference occurred more quickly for those in the presence condition than for those in the alone condition. This hypothesis is backed by the evidence that others’ presence increases interference at a perceptive earlier when participants perform the Ebbinghaus task, suggesting that in the presence of others, we are more sensitive to context (see Garcia-Marques et al., 2015). In performing an Ebbinghaus illusion-based task, those in presence showed greater size illusions than those in an alone condition, showing greater difficulty in perceiving the correct size of a target circle and ignoring its surroundings. Similar to the results obtained in our study, the delta plot functions suggested that this perception occurred in the initial stages of processing since when individuals took longer to respond, performance was as good as performance in the alone condition. Future studies should approach the possibility that oculomotor and perceptual behavioral responses can be assessing different cognitive mechanisms and that they may be differently influenced by others' presence. There are two decisions engaged in antisaccade performances, the decision of moving the eye (a top-down decision) and the decision of moving the hand (which may imply the bottom-up accumulation of information; see Carpenter & Williams, 1995; Carpenter, 2012). These two decisions may show different sensitivity to the presence of others.

These results suggest as likely that the two tasks are grasping different ways of how the presence of others modulates our cognitive processes. Although previous research suggested the overlap and reliability of interindividual differences in these two tasks and the Stroop task (e.g., Miyake & Friedman, 2012; Paap & Sawi, 2016; Rey-Mermet et al., 2018), other studies already suggest that Stroop, antisaccade and the stop signal do not similarly assess our control abilities (see Friedman & Miyake, 2017; Karr et al., 2018; Miyake & Friedman, 2012, for reviews). Future studies that would like to map the different cognitive mechanisms engaged in these tasks should take into account that although they can sometimes overlap (Miyake et al., 2000) they are likely to differ in details that show different sensitivity to others’ presence. Adding to this, are the effects promoted by using different versions of the tasks and the different measures associated with them which we previously discussed.

In sum, the differences in how interference is implemented may help to understand how control is organized when adjusting to errors in performance and why others’ presence modulates them differently or similarly. The cost of the inhibition task is likely dependent upon how interference is implemented in the tasks and how individuals may counteract it or correct their responses. The several models trying to understand both the Antisaccade task (see Munoz & Everling, 2004, for a review) and the stop signal task (see Verbruggen & Logan, 2008) should consider that others’ presence modulates them differently.

### Explaining the Presence of Other Effects

The effects of the presence of others around us have been historically considered social facilitation/inhibition effects (see Aiello & Douthitt, 2001). The social facilitation label has, however, become an umbrella for several phenomena that can occur when an individual is in the presence of others: evaluation, competition, social comparison, etc. Apart from that label, we focus on the effect that occurs simply when individuals perform a task alone versus in others’ presence, without the presence of the experimenter in the room or any other source of evaluation. This definition makes our approach to an effect that is more akin to the concept of the mere presence effect (Zajonc, 1965) and aims to question the effects of the minimal social setting factor (Sanders, 1981).

When Huguet et al. (1999) show evidence that presence conditions favor Stroop performance, they directly contrast a narrow attentional explanation of the presence of others effect (Baron, 1986) with Zajonc’s (1965) and Zajonc and Sales (1966) view that mere presence increases the use of dominant responses. The Zajonc view suggested that in a mere presence condition, participants use their well-learned responses better and faster when they are adapted to the current task. Thus, in inhibitory control tasks, we should expect interference to be higher for those in presence than for those in alone conditions, making control less efficient. A similar expectation would be derived from Allport’s (1920) spreading-out-of-thought account suggesting that responses of individuals in others’ presence conditions are more sensitive to the context (Fonseca & Garcia-Marques, 2013; Garcia-Marques et al., 2015; Liu & Yu, 2017; Puntoni & Tavassoli, 2007). In this case, interference should not only be higher in presence than alone conditions but would also be expected to occur quicker for those in presence conditions. The fact is that both in the Stroop task (Huguet et al., 1999) and the performance in the SST presented in this paper, control was exerted better in the presence of others, suggesting that if presence is increasing such susceptibility to interference, is not only doing it. Our data with the Antisaccade task are in line with the data of Allport and Zajonc given that performance is quicker in the presence of others, and the results show evidence that the interference was more quickly instated in the presence than in the alone condition. However, in another setting, with the signal detection task, our data suggest an increase in control abilities by showing that participants in that same presence condition were better performing the inhibition task. Therefore, and in line with all social facilitation research, these results suggest that both processes are occurring in presence relative to alone conditions: an increased sensitivity to what is accessible in our internal and external context and an increase in control ability.

This dual interference hypothesis would explain why the data are constantly corroborating the different explanations offered for the presence of other effects. This dual interference hypothesis translates a dynamic relationship between the activation + control mechanisms stating that the presence of others would increase both working memory sensitivity and the ability to avoid undesirable interferences that can occur as consequences of such activation. In contrast, when alone, the reduced sensitivity to context influences would not impose the need for an increased activation of control mechanisms. Future work should understand if this activation + control hypothesis can explain differences found in the performance of the Antisaccade task and the SST; namely, if tasks that allow the identification of an interference occurring earlier (likely signaling more) for those in presence rather than alone are also the tasks for which the increased control abilities of those in presence will allow them to have equivalent performance to those alone. However, tasks for which interference is not quickly installed allow better detection of the increase in control ability occurring in presence conditions. Currently, evidence suggests that both the Antisaccade task (current data) and the Ebbinghaus task (Garcia-Marques et al., 2015) are examples of the first type of task, and the SST (current data) and the Stroop task (see Garcia-Marques & Fernandes, under review) are examples of the second case.

## Notes

1 This restriction aimed to turn our sample more homogeneous with regard the baseline of participants executive function ability. Several studies have described age-related effects on the performance of this task (e.g., Butler et al., 1999; Munoz et al., 2004; Nieuwenhuis et al., 2000; Olincy et al., 1997). Butler et al., 1999; Nieuwenhuis et al., 2000; Olincy et al., 1997

2 Given that F <1, we do not report their *p* value because the *p* value associated with this analysis is meaningfulness. Statistical significance is impossible when F is smaller than 1 and so theoretically, we should consider *p* = 1 (see Voelkle, 2007).
